# A deep learning based system for handwashing procedure evaluation

**DOI:** 10.1007/s00521-022-07194-5

**Published:** 2022-04-21

**Authors:** Antonio Greco, Gennaro Percannella, Pierluigi Ritrovato, Alessia Saggese, Mario Vento

**Affiliations:** Department of Computer and Electrical Engineering and Applied Mathematics, University of Salerno, Fisciano, SA Italy

**Keywords:** Handwashing procedure, Deep learning, Depth camera

## Abstract

Hand washing preparation can be considered as one of the main strategies for reducing the risk of surgical site contamination and thus the infections risks. Within this context, in this paper we propose an embedded system able to automatically analyze, in real-time, the sequence of images acquired by a depth camera to evaluate the quality of the handwashing procedure. In particular, the designed system runs on an NVIDIA Jetson Nano$^{{\mathrm{TM}}}$ computing platform. We adopt a convolutional neural network, followed by a majority voting scheme, to classify the movement of the worker according to one of the ten gestures defined by the World Health Organization. To test the proposed system, we collect a dataset built by 74 different video sequences. The results achieved on this dataset confirm the effectiveness of the proposed approach.

## Introduction

Nowadays, about 722,000 patients yearly in the world are affected by a healthcare associated infection; among them, $10\%$ of the infected patients eventually die [1]. About $40\%$ of healthcare associated infections are caused by an improper hand hygiene among healthcare workers (hereinafter only workers), which clean their hands less than half of the time they should [2]. Thus, it becomes more and more important for the public safety the adoption of strategies to improve hand hygiene, so as to reduce the healthcare infection rates.

Within this context, it is particularly relevant the surgical hand preparation, aimed at minimizing the risk of surgical site contamination with microorganisms originating from the surgeon’s hands.

In 2009, the World Health Organisation (WHO) released guidelines on *Hand Hygiene in Healthcare* [3] to be adopted as a standard procedure for surgical hand washing; the procedure is composed of a sequence of well-defined gestures (detailed in Table 1) that the worker has to perform in a predefined order and with each gesture having a minimum time duration.

To evaluate the compliance with hand hygiene procedure, three main techniques can be adopted [3]: (1) *direct observation* of practice; (2) *self-report* of healthcare workers and (3) *indirect calculation*, based on the measurement of the products’ usage. According to Haas and Larson [4], *self-report* is not really accurate, while *indirect calculation* based on the measurement of products does not provide information of non-compliance. On the contrary, *direct observation* is considered the gold standard, since it provides all the information required for the analysis; unfortunately, this task is typically carried out by human observers, thus it is time-consuming and expensive.

To reduce the costs associated with *direct observation*, in the last years some automatic or semi-automatic solutions to allow a machine to guarantee direct observation during the hand-washing [5] have been proposed. For machine-based *direct observation*, it is required to have a system that can (1) monitor the compliance with hand hygiene procedure and also (2) provide a real-time feedback to the worker during the hand washing procedure, allowing workers to improve the gestures and then reduce the risk of infection for the patient.

Among the most promising methods, video analytic has surely played a key role. Indeed, one of the most important milestones in this field has been the introduction of camera sensors for data acquisition, as well as the development of algorithms based on artificial intelligence, typically traditional machine learning, for the automatic analysis of the acquired images and videos [6] [7] [8]. Nevertheless, the literature is still quite limited, and a definitive solution to the problem has not been found yet.

In this paper, we propose a method based on deep learning for monitoring the surgeon handwashing procedure. The proposed system has been designed to be applied to both younger staff training and surgeon hand washing evaluation. Indeed, it is able to analyze the sequence of images acquired by a depth sensor and to classify the gestures performed by the worker in real-time. Moreover, the proposed method can provide a compliance feedback score, with respect to the single gesture, in terms of compliance with the guidelines. The continuous feedback about any single gesture allow the worker to realize what the actual performance are, so as to immediately adapt the movement in case of low conformance visual feedback. This approach is particularly relevant during the training phase, allowing the trainees to immediately recognize possible errors, but also to engage surgeon to respect the procedure timely.

With respect to state-of-the-art, we introduce two main novelties: (1) we propose a novel method for continuous surgeon handwashing procedure evaluation based on deep learning; the gestures are analyzed and classified by means of a Convolutional Neural Network; furthermore, the temporal information is taken into account *via* an overlapped sliding window; indeed, the decision is not taken by evaluating the single frame, but instead by considering a sequence of frames with a majority voting rule; (2) we built a dataset composed of 74 different video sequences; the dataset contains the sequence of gestures officially defined by the *Hand Hygiene in Healthcare* guidelines; the dataset is freely available under request for benchmark purposes^1^; according to our knowledge, this is the first dataset on this topic made publicly available.

The paper is organized as follows: Sect. 2 introduces related works. In Sect. 3, we detail the proposed system fd98, together with the experimental setup and the description of our dataset. In Sect.  5, we define the metrics used in our experimentation and report the obtained results. Section  4 describes how the system works. Finally, we draw some conclusions and future work in Sect.  6.

## Related work

Among the methods proposed in the last years for direct observation, we can identify two main approaches, depending on the type of sensors used for the measure. In the first category we can identify the systems employing a visual sensor and a video analytic algorithm that automatically analyze the gestures performed by the worker during the hand-washing procedure [9]. The second category includes those systems adopting other kind of sensors, typically wearable, such as smart watches [10]. Harmony [11] is an example of system belonging to the latter category. It is a hand wash monitoring system based on distributed sensors: each worker wears a smart watch, able to collect information related to linear acceleration, gravity, and gyroscope signals. Furthermore, a set of Bluetooth devices is put close to soap dispensers and in areas of interest, such as wash zones and patient bed zones. The smart watch communicates with sensors for dynamic activation and deactivation and analyzes the movement of the worker so as to identify and evaluate her/his gestures. The system is invasive and potentially expensive, since it requires the introduction of a smart watch for each worker to be monitored. Even if this is effective for generic healthcare workers, it can not be worn by surgeons, due to hygiene rules inside the operating rooms. Thus, we do not consider this as well as other similar approach like [12] as feasible for our purposes.

An interesting system is *RFWash* [13]. In the paper, authors propose use of a radio-frequency (RF) commercial-off-the-shelf mmWave sensor for evaluating the 9 gestures the WHO recommended for alcohol-based handrub. The authors characterize the challenges of recognizing back-to-back hand gestures using an RF-based gesture recognition processing pipeline. Indeed, as evident, the lack of pauses between gestures makes segmentation difficult, which, in turn, affects the performance of the subsequent classification component. For that reason a new sequence learning approach that performs segmentation and recognition simultaneously has been proposed. The model is trained using continuous stream of minimally labelled RF data corresponding to naturally performed handrub gestures. The RFWash performance have been carried out using a dataset of 1,800 gesture samples collected from ten subjects over 3 months. A deep architecture has been defined including Convolution layers followed by Max Pooling (2x2), Fully Connected (FC) and Bidirectional LSTM layers, aiming at extracting spatiotemporal gesture features from input RD frames; also, a softmax layer and Connectionist Temporal Classification (CTC) is employed to predict the gesture sequence. From the performance point of view, with sequences of a duration of 5s per gesture the mean gesture error rate (GER) is about 11% while increasing the duration to 10s per gesture the mean GER drop to 7.41%.

In [14], a system for the automatic analysis of the hands after the washing has been proposed: the hands are washed with soap mixed with UV reflective powder; the operator has to insert the hands inside a case equipped with ultraviolet lighting and a digital camera. The presence of ultraviolet lighting leads the skin to show ultraviolet light only on the treated surfaces. The images acquired by the camera are then automatically analyzed by a segmentation algorithm, and the contour of the hand is determined by evaluating the green intensity channel. The green channel pixels belonging to the hand are partitioned in three clusters using c-means clustering algorithm. The optimal threshold between the intensity of clean and dirty areas is extracted using these clusters, to evaluate a percentage of dirty area and the remaining percentage of clean area. Even if based on a camera and on visual inspection, the system, like the previous one, is still quite invasive, since it requires the adding of UV reflective powder in all the soap dispensers; furthermore, the introduction of the UV-lighting case in the equipment could be a further source of contamination, infringing hygiene hand-washing rules for operating rooms.

In [8] a camera sensor put on top of the washing machine is introduced and a video analysis algorithm is proposed to automatically evaluate the quality of the washing procedure. This is among the first methods in which the sequence of gestures performed by the surgeon has been automatically analyzed, thanks to the introduction of a machine learning approach. Indeed, the segmentation step combines information related to both color and motion; then, a tracking procedure based on a single multi-modal particle filter and a k-means-based clustering technique is adopted to track both hands and arms. Finally, a SVM ensemble classifier has been employed for recognizing the specific gesture. The use of a traditional camera introduces several issues related to variation in illumination conditions, as well as to the presence of the water. The experimentation has been carried out on 6 different hand poses with detection rates performance ranging from about 86% up to about 97%.

**Fig. 1:**
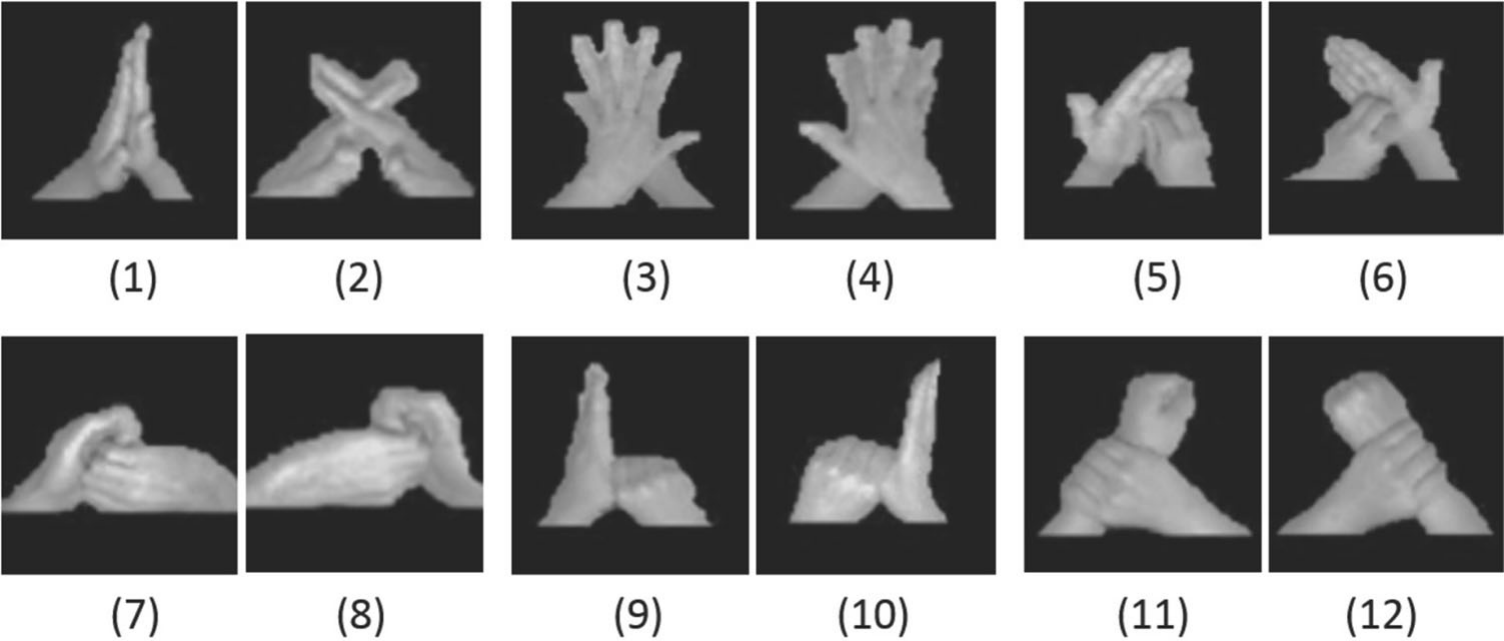
the 12 poses used in [6]

In [6], Xia et al. improved the performance achieved by [8] extending the number of poses to recognise from 6 to 12 (using poses for left and right hands see 1) adopting a SoftKinetic DS325 camera and applying Linear Discriminant Analysis (LDA) classifier. The performance on single frame pose estimation has been evaluated using a Leave-One-Person-Out (LOPO) subject-independent cross-validation protocol, considering both RGB and depth images. To address the high dimensionality of the HOG (Histogram of Gradient) features (2916 dimensions), in each round of the cross-validation the original HOG features are projected in lower dimensional subspace using the Principal Component Analysis (PCA). According to their analysis the LDA classification cost about 0.0139 ms on an Intel i7 with a 3.70 GHz clock. The recognition rate on single frame have been of 94.80% on RGB channel and 92.35% for depth channel. Finally a further experiment has been executed considering a video-pose estimation with a slide windows of different sizes and using a majority voting on single frame classification. With a windows size of 20 frames the achieved recognition rates are 99.37% and 98.31% for the two channels respectively. Authors declared that the recognition rate is 100% when the size of the windows is equal to whole video of the single poses (Fig. 1).

Many of the proposed solutions requires computing powers such one provided by modern PC and those based on RGB cameras, due to the different environmental condition present tuning and setup problems when installed in different surgery blocks that prevent their application in real context. In [7] the authors use a depth sensor, namely a Kinect sensor, instead than a traditional camera. The system they propose, called *WashInDepth*, is able to record the washing procedure and determine if the subject has correctly complied with the prescribed guidelines. A background subtraction is applied and a set of hand-crafted features is extracted and then used to fed a decision tree classifier. The experimentation has been carried out with the involvement of 15 participants for two different scenarios. in the person independent scenario (where data from 10 participants were used for training and 5 for testing) the best performance achieved has been of about 55%. In the person depended scenario (Wherein, both the training and test data is from the same person) the best achieved performance has been of about 97%. Both performance have been achieved with a 15 $\times$ 15 block size and smoothing windows of length equal to 50. Another important aspect of the *WashInDepth* solution is the possibility to run an a computer stick.

This kind of approaches, exploiting camera and video analytics solutions, represent a very important milestone in the scientific literature in this field. Anyway, although the topic is very relevant, as demonstrated by the main recommendation from the WHO for fighting the recent coronavirus pandemic, we can not find a wide literature; this is probably due mainly to the lack of dataset publicly available to be used for training, but also for benchmarking purposes. Indeed, in the era of deep learning, a huge amount of data becomes essential [15].

To face with this issue, we can surely inherit the wide literature available in gesture recognition [16–19]: indeed, each movement to be performed from the worker can be seen as a specific class of gesture to be recognized and analyzed. Within this context, the deep learning plays a crucial role, since most of the algorithms proposed in the last few years are based on this new frontier of artificial intelligence. Although there is not a standard taxonomy for partitioning the methods for gesture recognition, we can identify two interesting contributions, proposed respectively in [20] and [21]. Their taxonomy is mainly based on the type of sensor used for analysing the movement: vision-based [22], glove-based [23] and depth-based.

According to the above mentioned surveys, the first two approaches are not promising and natural enough, while the most promising methods available in the literature are based on depth cameras, which allows to exploit the third dimension related to the depth. This conclusion is still valid in our specific problem; indeed, the gloves can not be used for hygiene reasons and the vision based system suffers for environmental conditions; vice-versa, depth sensor, able to also evaluate the three dimensional space, seems to be the most suited sensor.

Independently on the sensor adopted for acquiring the set of images, the best results in gesture recognition are typically obtained by using convolutional neural networks (CNNs), which achieve outstanding results outperforming “non-deep” state-of-the-art methods [21]. Although a lot of different CNNs has been proposed in the last years [24, 25], the new trend seems to be mainly related to the introduction of recurrent neural network (RNNs), such as LSTM, GRU or TCN [26, 27], able to automatically encode the temporal information, which is evidently a very important and not negligible feature when dealing with gestures evolving during the time.

Anyway, the main drawback lies in the amount of data required for training when dealing with RNNs, which is typically higher with respect to the CNNs counterpart. This is an important consideration, since in our specific problem the amount of data is quite limited.

## Methods and materials

### Dataset

The sequence of specific gestures to be performed during the hand washing procedure depends on several factors, including the context (e.g., patient care, visit, surgical operation), the type of soap, the use of specific tools like nail cleaners or sponges and so on. In this paper, we focus on the surgical hand washing procedure, as described in [28]. The procedure includes eleven different gestures, which need to be performed in a given order; the details of each gesture are reported in Table 1, together with an abbreviation of the gesture itself, which will be used hereinafter in the paper. Gestures 1 and 11 are exactly the same.

**Table 1:**
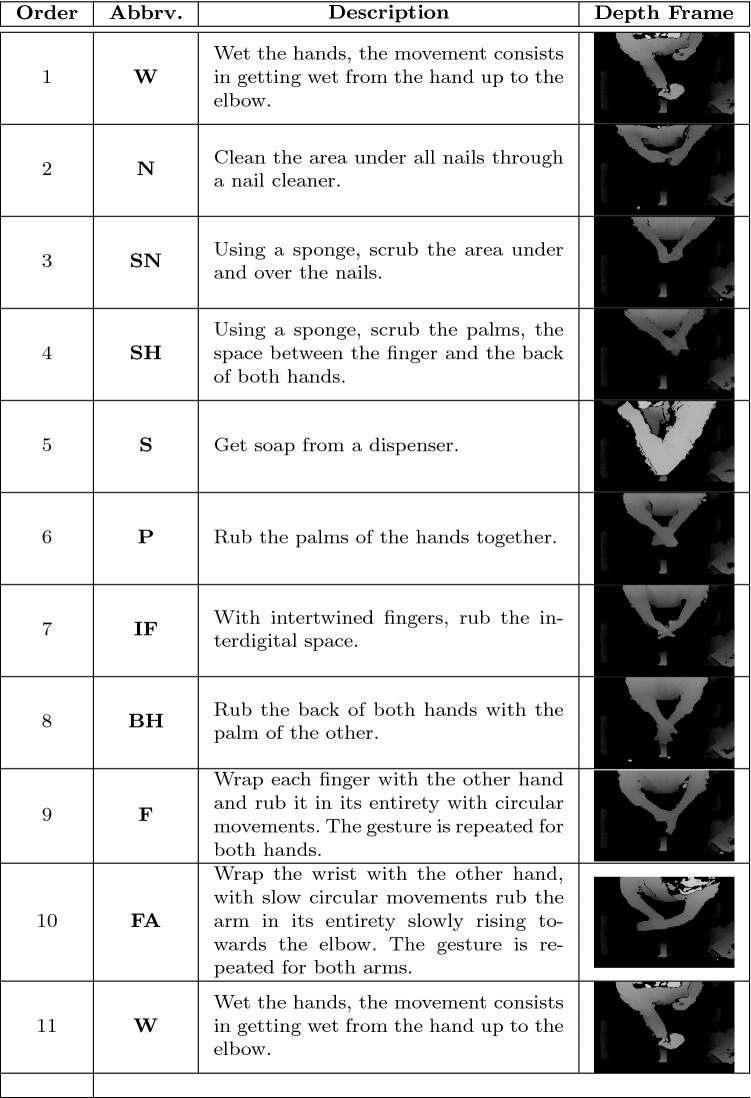
Gestures of the surgical handwashing procedure

For each gesture, we report the position in the sequence, the abbreviation used in the paper, a brief description and a representation picture. The 11st gesture is the repetition of the first one

The dataset we propose was collected with the support of professors from the Department of Medicine, Surgery and Dentistry - “Schola Medica Salernitana” of the University of Salerno, Italy.

The procedure was simulated by 53 different voluntaries, equally distributed between males (27) and females (26). The participants also had different height, so implying that the procedure was performed at different distances with respect to the camera. All the participants signed an informed consent. Each voluntary was properly trained by a medical doctor before performing the procedure; furthermore, during the procedure itself, a video with the specific gesture to be performed was shown to the worker (see Sect.  4 for more details). Also, each sequence was validated by a doctor before its insertion into the dataset.

The camera is mounted at a height above the washbasin of $D_{plane} = 0.9m$, in a zenithal position; the top view allows for the movements of the hands and of the arms without any occlusions; furthermore, the chosen height also entirely captures the area where the worker has to move for washing his/her hands.

The camera used for the acquisition of the dataset is an Intel^®^ RealSense$^{{\mathrm{TM}}}$ Depth Camera D435. The camera is controlled by a NVIDIA^®^ Jetson Nano$^{{\mathrm{TM}}}$ computing platform equipped with Quad-core ARM^®^ Cortex$^{{\mathrm{TM}}}$-A57 CPU, a NVIDIA Maxwell$^{{\mathrm{TM}}}$ with 128 core NVIDIA CUDA^®^ GPU and 4GB LPDDR4 64-bit of RAM running Ubuntu operating system. The system was used for building the dataset and for interpreting the worker gestures providing real-time feedback using the designed GUI. The dataset consists of 74 depth video sequences; each video contains the sequence of the ten gestures, obtained by a continuous capture of the whole hand washing procedure performed by a worker. The depth images are represented in 16 bits, where each pixel represents the distance from the camera (in millimeters). Each image is captured at a resolution of $640 \times 480$, and the acquisition is performed at 15 frames per second. Sample depth images for each gesture are shown in the rightmost column of Table 1.

The dataset was partitioned into training and test set. The training set is composed of 50 sequences recorded by 41 different subjects; the test set includes the remaining 24 sequences, performed by 12 different subjects. In the whole, the dataset consists of more than 131, 000 frames, as reported in Table 2. The table also reports for each gesture the number of frames and the average duration. We can note that the average duration of the gestures ranges from short gestures (4 seconds), such as *W* and *S*, to long gestures (more than 20 seconds), such as *N* and *SH*.

**Table 2:** Overview of the dataset

**Gesture**	**Train set**	**Test set**
	*/# of frames / duration*	*/# of frames / duration*
**W**	3236 / 4s	1448 / 4s
**N**	17,057 / 23s	5744 / 16s
**SN**	10,850 / 14s	3906 / 11s
**SH**	16,198 / 22s	6096 / 17s
**S**	3149 / 4s	1109 / 3s
**P**	6645 / 9s	2943 / 8s
**IF**	6424 / 9s	2856 / 8s
**BH**	14,000 / 19s	5579 / 15s
**F**	7492 / 10s	2886 / 8s
**FA**	10,464 / 14s	3683 / 10s
**Total**	95,515 / 128s	36,250 / 100s

For each gesture, the number of frames and the average duration of each gesture is provided for both training and test sets

### Proposed method

In this paper, we formulate the problem of assessing the conformance of the hand washing procedure in terms of a gesture recognition problem. In more details, we train a classifier to associate the frame to one of the ten classes, each class being associated to a gesture.

Each image is cropped to $340 \times 340$, so as to only deal with the central region of the image, containing the hands of the worker. Furthermore, a bicubic interpolation is applied for rescaling the image to $170 \times 170$. To only isolate regions of interest and to remove the background from the analysis, we apply a threshold on each image; with more details, we consider as background all the pixels in the image at a distance higher than $D_{th}$, where $D_{th} = D_{plane} - \epsilon$. In our experiments, $\epsilon = 1cm$. Finally, we halve the size of samples (from 16 bits to 8 bits) and rescale the values as follows:

1 $$\begin{aligned} s_{r}(i,j) = {\left\{ \begin{array}{ll} M\cdot (1-\frac{s_{o}(i,j)}{D_{th}}) &{} \text { if } 0\le s_{o}(i,j)\le D_{th} \\ 0 &{} \text {otherwise} \end{array}\right. }, \end{aligned}$$

where $s_{o}(i,j)$ and $s_{r}(i,j)$ are the values of the (*i*, *j*)-th pixel of the image before and after rescaling, respectively.

The images preprocessed as described above are fed to a classier. Five different architectures are considered in our experimentation, namely VGG19 [29], ResNet50 [30], Xception [31], MobileNet [32] and NASNet [33]. The architectures are chosen to take into account both different dimensions and different typologies of layers. In particular, we considered: (1) networks of different dimensions, namely large (VGG19, NASNet), medium sized (ResNet50, Xception) and small networks (MobileNet); (2) networks based on different concepts, from traditional convolutional layers (VGG19, NASNet) to more modern blocks inspired by Network-In-Network architectures, respectively based on either residual blocks (ResNet50) or on depthwise separable convolutional layers (Xception, MobileNet).

**Fig. 2:**
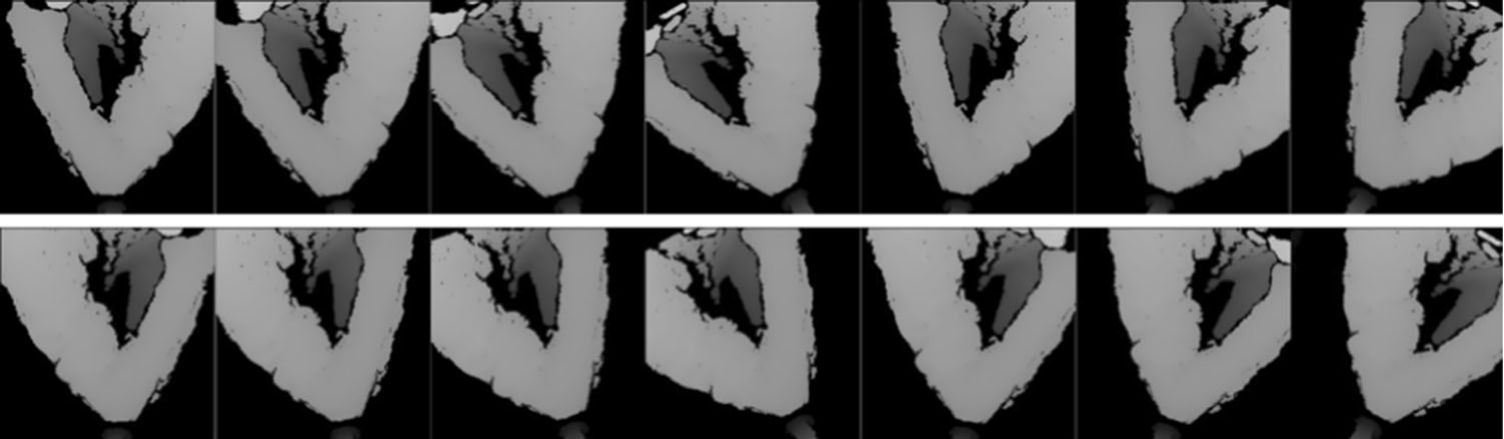
Example of images generated after data augmentation. The first column contains the original image (top) and the flipped version (bottom). From the second to the last column, we can find rotated versions of the first image; from left to right, counterclockwise and then clockwise by $10^{\circ }$, $20^{\circ }$ and $30^{\circ }$

During the training procedure, independently of the specific architecture considered, we applied a data augmentation technique to increase the robustness of the method with respect to the following main situations (Fig. 2): (1) the worker can be either right-handed or left-handed; (2) while washing the hands, the worker could assume oblique position with respect to the washbasin. Starting from this consideration, we augmented the dataset with (1) flipped and (2) rotated images. The rotation was performed by $10^{\circ }$, $20^{\circ }$ and $30^{\circ }$ in both directions. We did not consider larger rotations since they cannot be physically done by the worker.

## How the system works

**Fig. 3:**
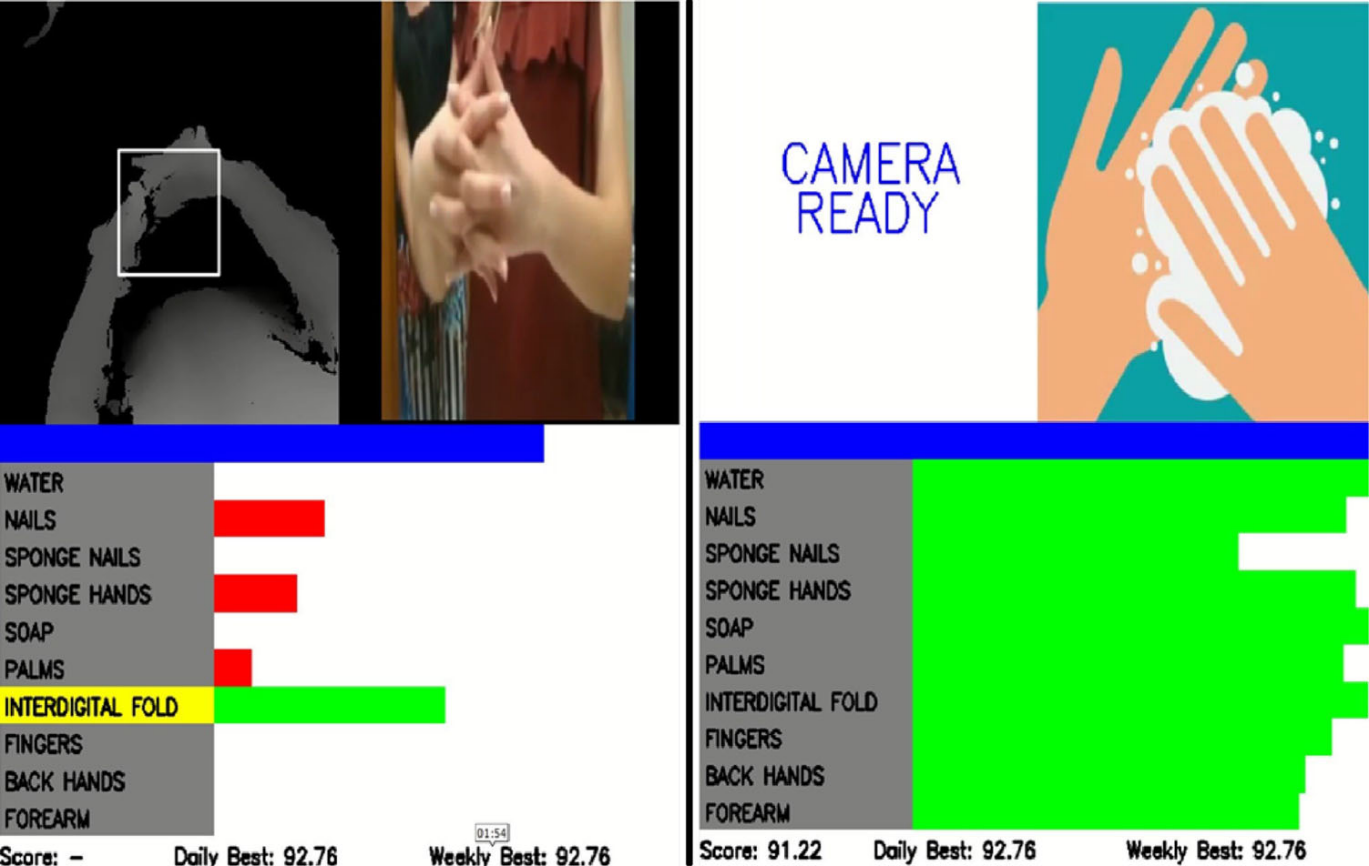
Handwashing system GUI: Real-time feedback (Left); Overall performance (right)

The system was designed to work in real-world environments. To design the graphical user interface (GUI) of the system, we started with an observation period of medical staff in the frame of the BIPS national research project. During this observation period, we noted medical staff habits and discussed with them several possible GUI alternatives. The most appreciated is the one presented in Fig. 3. An automatic activation function was developed to facilitate the access to the system: it is required to maintain the hands under the camera for at least 3 s and the system starts.

The GUI is divided into three areas. Going in clockwise order, in the upper-left corner area the real-time video of the deep camera is showed. For facilitating the gesture recognition, a white box is impressed over the video, so that the medical staff can easily center his/her hands under the camera at the right distance. In the upper-right corner, a recorded video of the actual gesture to execute according to the WHO procedure is presented.

The bottom-half of the GUI is dedicated to the real-time system feedback. The area is divided into twelve rows. The first row, marked in blue, is a progress bar that indicates the remaining time for completing the actual gesture. This is important since each gesture has a specific duration as defined by the WHO guidelines; the next tens rows provide the real-time gestures recognition feedback and finally the bottom row report the performance: the actual achieved score (updated at the end of the procedure), the daily and weekly best scores. This gaming part was appreciated by the medical staff.

The most relevant part of the GUI is the one dedicated to the single gesture feedback. Differently from what the other systems offer, exploiting the system architecture based on ten different classifiers and the selected hardware platform, our solution can show in real-time the level of classification of the gesture actually recognised by the system. To advise the medical staff with the gesture to be performed, its name is highlighted in yellow (e.g., in Fig. 3 the *Interdigital fold* has to be executed). The level of compliance of the gesture is provided by a green bar: the longer the bar, the higher is the compliance with the gesture described by the WHO guidelines (full length mean $100\%$ compliance).

The system provides a visual feedback about misclassifications too. Indeed, during the specific gesture execution, the GUI shows (through red bars) the level of classification of the other gestures. Since this happen in real-time, the user immediately realises that the performed gesture is not properly recognised by the system and try to improve its execution in the remaining time. Also this feature was considered very useful from the medical staff.

Finally, when all the gestures were executed, the overall performance is summarised by the GUI (right part of Fig. 3) so that the medical staff next time can improve the execution of those gestures with a lower level of recognition. The Score value is calculated as the average of the estimated accuracy of the gestures.

## Experimental results

In this section, we present the performance achieved by the proposed system for conformance assessment of the hand washing procedure carried out by an healthcare worker. We assess the impact on performance of the different choices made during the design phase of the proposed approach by considering two main dimensions of analysis: the deep network architecture and the temporal dimension exploitation.

As regards the first point, we consider different state-of-the-art deep neural network architectures and for each of them we evaluate performance using different optimizers, loss functions and initialization procedures. Then, we select the network architecture and configuration providing the highest performance on the test set as the base network for the successive studies related to the remaining dimension of analysis.

As for the second point, we explore the beneficial impact that may derive from the exploitation of the temporal information, which allows us to make the classification decision on a sequence of contiguous frames instead of a single frame. In this direction, we consider the aggregation by majority voting, or weighted voting, of the outputs provided by a classifier on all the frames within a sliding window.

The remainder of the current section is organized as follows: in Sect. 5.1 we describe the indices used to measure performance; then, we dedicate a specific subsection to each dimension of analysis: the deep network architecture and the temporal dimension exploitation are discussed in Sects. 5.2 and 5.3, respectively.

### Performance indices

Depending on the level of detail and the goal of the analysis, we use several indices to assess the performance achieved by the investigated methods. In particular, the $f_1$ score is adopted as a single global performance index that compares and ranks different solutions; we also report the *Precision* and *Recall* to provide additional information on the type of the errors, i.e., false positive and false negative, respectively. The above three indices are calculated as:

2 $$\begin{aligned}&f_1 = \frac{1}{\sum _{l \in L} \left| \hat{y}_l\right| } \sum _{l \in L} \left| \hat{y}_l\right| F_1(y_l, \hat{y}_l) \end{aligned}$$

3 $$\begin{aligned}&Recall = \frac{1}{\sum _{l \in L} \left| \hat{y}_l\right| } \sum _{l \in L} \left| \hat{y}_l\right| R(y_l, \hat{y}_l) \end{aligned}$$

4 $$\begin{aligned}&Precision = \frac{1}{\sum _{l \in L} \left| \hat{y}_l\right| } \sum _{l \in L} \left| \hat{y}_l\right| P(y_l, \hat{y}_l) \end{aligned}$$

where:

5 $$\begin{aligned} F_1(y_l, \hat{y}_l)= & {} \frac{2 \cdot P(y_l, \hat{y}_l) \cdot R(y_l, \hat{y}_l)}{P(y_l, \hat{y}_l) + R(y_l, \hat{y}_l)}, \nonumber \\ P(y_l, \hat{y}_l)= & {} \frac{\left| y_l \cap \hat{y}_l \right| }{\left| y_l\right| }\;\;\; \text {and}\;\;\; R(y_l, \hat{y}_l)\nonumber \\= & {} \frac{\left| y_l \cap \hat{y}_l \right| }{\left| \hat{y}_l\right| } \end{aligned}$$

*L* is the set of the labels, *y* and $\hat{y}$ are the sets of *predicted*(*sample*, *label*) and *true*(*sample*, *label*) pairs, respectively, $y_l$ and $\hat{y}_l$ the subsets of *y* and $\hat{y}$ with label *l*.

**Fig. 4:**
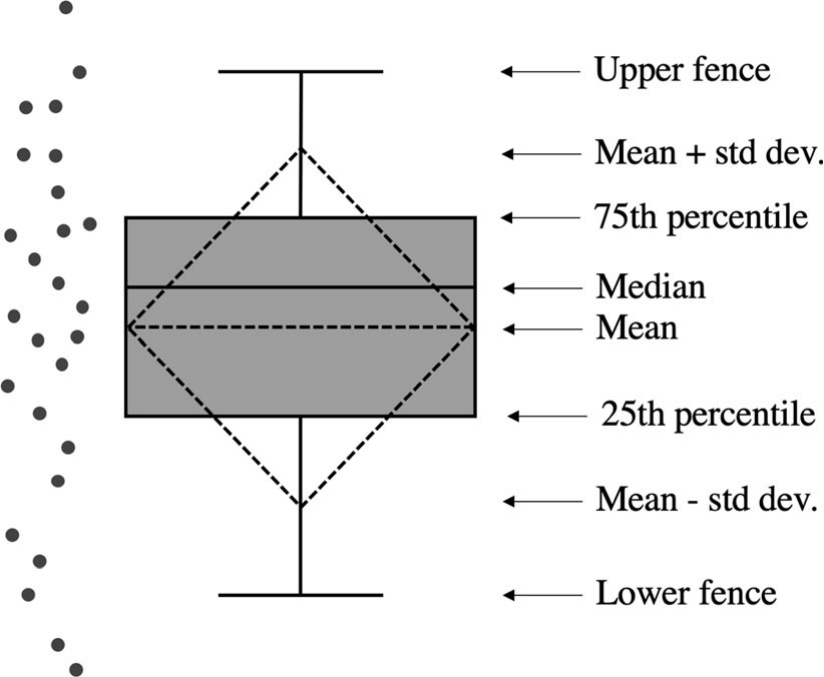
Elements of the box plot model used for reporting compactly the salient elements of a distribution: they report the 25th, 50th (median) and 75th percentiles, the mean value and standard deviation. The upper and lower fences represent values more and less than 75th and 25th percentiles (3rd and 1st quartiles), respectively, by 1.5 times the difference between the 3rd and 1st quartiles; values above upper fence or below lower fence are generally declared as outliers

We also use the box plots (see Fig. 4 to briefly recall the elements of the box plot representation) as a tool for studying the impact over performance when setting specific values for the various parameters of the deep networks. In particular, we use this representation to graphically depict the collection of values of the $f_1$ score that are obtained by setting a value for a specific method parameter and by varying all the remaining parameters.

Finally, we use the $10 \times 10$ confusion matrix for reporting the performance for each gesture class to focus on the ability of the system to distinguish some hand gestures from others.

### Deep network architecture

To select the classifier used as base for the successive design stage regarding the exploitation of the temporal information, we consider various possible choices of the deep network architecture, the optimizer, the loss function, the training initialization procedure. In particular, as for the deep network architecture, we consider **MobileNet**, **NASNet**, **ResNet50**, **VGG19** and **Xception**. As optimizer, we consider the following four choices: **Adam**, **Adadelta**, **SGD**, **RMSprop** [34]. We investigate also on the loss function by considering the mean square error (**MSE**) and the categorical cross entropy (**CCE**). As a final element of the study, we consider the starting point of the training procedure, i.e., training the network from scratch using randomly initialized weights (**R**) or, conversely, using the weights of the corresponding network already trained on a different domain (in this case, we used ImageNet initialization - **I**).

In our tests, we consider all the possible quadruples of values given by the Cartesian product of the sets of the dimensions of analysis: we evaluate 80 models (5 network architectures $\times$ 4 optimizers $\times$ 2 loss functions $\times$ 2 training procedures). Each model is individually trained by starting with an initial value of the learning rate of 0.001 and then by decreasing it by a 0.3 factor when the validation loss does not increase after six consecutive epochs.

**Table 3:** Performance achieved over the test set by each of the 48 considered model configurations

**Arch.**	**Init.**	**Opt.**	**L.F.**	**Re**	**Pr**	**f1**
MobileNet	R	Adadelta	CCE	0.797	0.802	0.796
			MSE	0.806	0.810	0.806
		Adam	CCE	0.831	0.841	0.832
			MSE	0.847	0.856	0.849
		RMSprop	CCE	0.781	0.802	0.782
			MSE	0.833	0.847	0.835
		SGD	CCE	0.764	0.778	0.767
			MSE	0.737	0.758	0.740
	I	Adadelta	CCE	0.877	0.881	0.878
			MSE	0.851	0.850	0.849
		Adam	CCE	0.851	0.856	0.852
			MSE	0.834	0.841	0.832
		RMSprop	CCE	0.838	0.849	0.839
			MSE	0.873	0.875	0.873
		SGD	CCE	0.797	0.805	0.798
			MSE	0.781	0.780	0.778
NASNet	R	Adadelta	CCE	0.802	0.803	0.801
			MSE	0.767	0.768	0.763
		Adam	CCE	0.804	0.817	0.804
			MSE	0.811	0.821	0.812
		RMSprop	CCE	0.752	0.751	0.749
			MSE	0.787	0.809	0.790
		SGD	CCE	0.794	0.792	0.792
			MSE	0.805	0.811	0.806
	I	Adadelta	CCE	0.836	0.837	0.835
			MSE	0.828	0.833	0.829
		Adam	CCE	0.838	0.840	0.837
			MSE	0.821	0.829	0.821
		RMSprop	CCE	0.795	0.795	0.793
			MSE	0.850	0.857	0.850
		SGD	CCE	0.811	0.813	0.811
			MSE	0.783	0.787	0.784
ResNet50	R	Adadelta	CCE	0.836	0.839	0.837
			MSE	0.828	0.829	0.827
		Adam	CCE	0.708	0.722	0.709
			MSE	0.699	0.704	0.697
		RMSprop	CCE	0.702	0.711	0.700
			MSE	0.711	0.740	0.715
		SGD	CCE	0.799	0.800	0.796
			MSE	0.807	0.805	0.805
	I	Adadelta	CCE	0.845	0.845	0.844
			MSE	0.855	0.856	0.855
		Adam	CCE	0.825	0.834	0.826
			MSE	0.813	0.815	0.813
		RMSprop	CCE	0.807	0.810	0.806
			MSE	0.827	0.831	0.826
		SGD	CCE	0.831	0.831	0.830
			MSE	0.781	0.791	0.779
**VGG19**	R	Adadelta	CCE	0.734	0.744	0.734
			MSE	0.822	0.826	0.822
		Adam	CCE	0.810	0.814	0.809
			MSE	0.751	0.768	0.751
		RMSprop	CCE	0.799	0.807	0.800
			MSE	0.791	0.803	0.793
		SGD	CCE	0.684	0.693	0.685
			MSE	0.819	0.821	0.818
	**I**	Adadelta	CCE	0.835	0.835	0.834
			MSE	0.806	0.808	0.805
		**Adam**	CCE	0.874	0.876	0.873
			**MSE**	**0.885**	**0.887**	**0.884**
		RMSprop	CCE	0.867	0.872	0.867
			MSE	0.833	0.842	0.832
		SGD	CCE	0.792	0.794	0.791
			MSE	0.753	0.769	0.754
InceptionV3	R	Adadelta	CCE	0.780	0.781	0.778
			MSE	0.764	0.773	0.763
		Adam	CCE	0.814	0.819	0.815
			MSE	0.757	0.766	0.755
		RMSprop	CCE	0.791	0.796	0.791
			MSE	0.793	0.796	0.792
		SGD	CCE	0.658	0.665	0.654
			MSE	0.719	0.731	0.717
	I	Adadelta	CCE	0.876	0.880	0.877
			MSE	0.837	0.844	0.838
		Adam	CCE	0.859	0.863	0.859
			MSE	0.835	0.838	0.836
		RMSprop	CCE	0.835	0.837	0.835
			MSE	0.848	0.850	0.848
		SGD	CCE	0.834	0.842	0.834
			MSE	0.836	0.838	0.836

The four leftmost columns account for the network meta-parameters, where specifically: **Arch.** stands for the deep network architecture, **Init.** stands for weight initialization (with R = random, I = from ImageNet), **Opt.** denotes the optimizer, and **L.F.** is the loss function (CCE = categorical croos-entropy, MSE = mean square error). The performance achieved by each model are in the three rightmost columns and are expressed in terms of Recall (**Re**), Precision (**Pr**) and *f*1. The model that achieves the highest value of *f*1 over the test set is highlighted in bold

In Table 3, we report the performance achieved on the test set by the considered models with indication of the respective configuration of the parameters. Specifically, the performance of each model is reported in the three rightmost columns of the table and are expressed in terms of the *Recall*, *Precision* and $f_1$ indices.

We notice a large variability of the performance of the different configurations of the classifiers ranging from a minimum value $f_1 = 0.654$ obtained by InceptionV3 using random weight initialization, using the SGD optimizer and categorical cross-entropy loss function, to the maximum value $f_1 = 0.884$ achieved by VGG19 architecture, initialized with ImageNet weights, using the Adam optimizer and the mean square error loss function (reported in bold in Table 3).

**Fig. 5:**
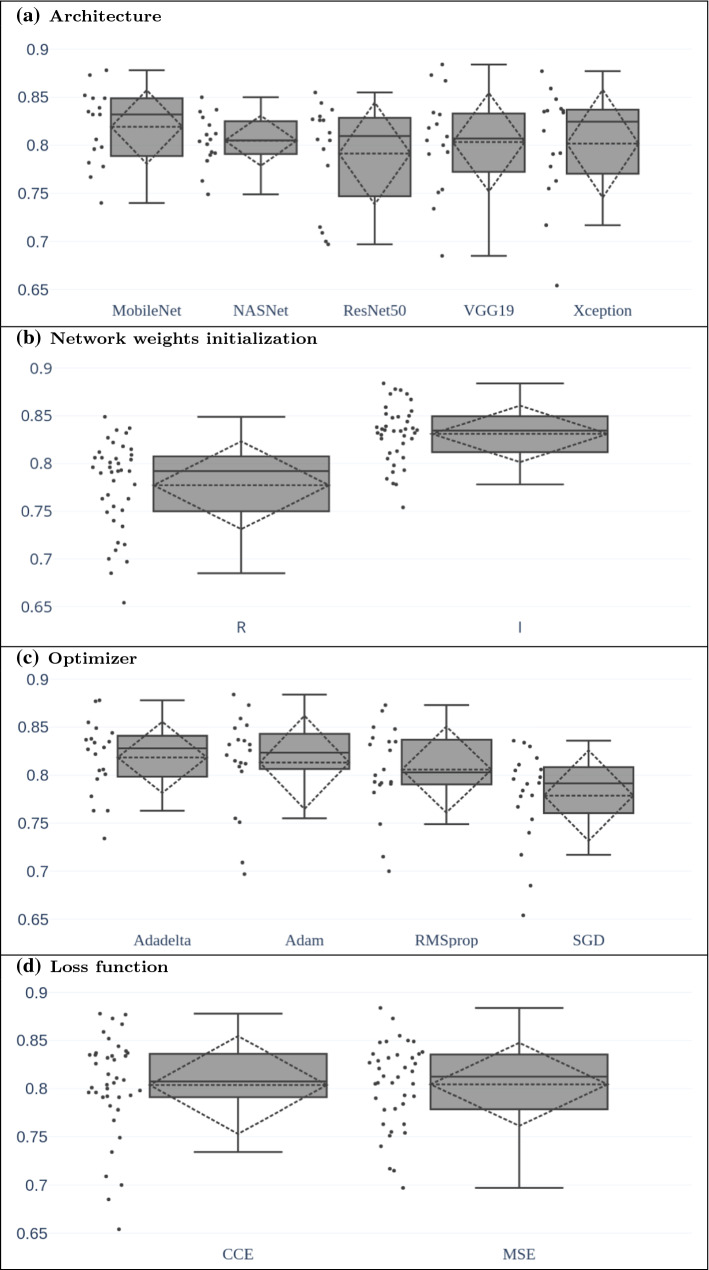
Representation by using box plots of the distributions of the *f*1 score for each dimension of analysis with respect to the remaining ones

The large variability of the results does not allow us to find evident correlations between the classification performance and each of the meta-parameters described above. To overcome this limitation, we use the box plots to compare the distributions of the $f_1$ score obtained by setting a value for each parameter and varying the remaining ones. Consequently, each plot of Fig. 5 deepens the study with regard to the architecture, the weight initialization, the optimizer and the loss function. As an example, the leftmost box plot in Fig. 5.a is derived from the 16 values of the $f_1$ score reported in Table 3 using the MobileNet as network architecture.

The first observation that we can draw from the analysis of the plots in Fig. 5 is that the weights initialization procedure is the only configuration parameter that has a relevant impact over performance. Fig. 5b suggests a superiority of the **I** choice with respect to **R**; this is further confirmed by the fact that 35 over 40 models (almost $90\%$ of cases) trained with weights initialized from ImageNet achieve an $f_1$ score superior to the corresponding model trained with randomly initialized weights. Conversely, for all the other meta-parameters we do not find a value that relevantly stands alone over the others with respect to the value of the *f*1 index.

Nevertheless, from Fig. 5a, we notice the high compactness of the box plot related to the NASNet, which denotes a degree of robustness of this network with respect to the considered training parameters that is higher than the other deep networks; in fact, with exception of NASNet, all the other networks are characterized by a large difference between the upper and the lower fences. From a practical standpoint, this may be a relevant aspect when one does not want to train the network by considering all the possible parameters configurations.

From Fig. 5c, we notice that on one side it has to be expected similar performance when choosing Adadelta, Adam and RMSprop while on the other side, at least for the problem under analysis, the SGD optimizer should be avoided.

Finally, Fig. 5.d does not suggest any particular advantage in using the categorical cross entropy or the mean square error as loss function for the problem under consideration.

### Temporal dimension exploitation

In this subsection, we analyze how the performance can be improved by exploiting the temporal dimension. The hand gesture occurs over a time interval and thus consists of a sequence of consecutive and highly correlated frames. The approaches considered in Sect. 5.2 take a decision over the generic *i*-th frame only using information extracted from that frame. Here, we intend to perform classification of the *i*-th frame by exploiting information of the *r* consecutive frames in the time window preceding the *i*-th frame.

There is a wide literature on methods for frame sequences analysis applied to gesture recognition. In this regard, the recent trends of the scientific community suggest the use of deep learning methods specifically devised to learn a time-series representation, as recurrent neural networks (RNNs). Long Short Term Memory recurrent networks (LSTM-RNN) represents a notable example of such approaches. However, it is well-known that such a method requires very large train datasets to achieve an acceptable level of generalization. This observation was also confirmed by experiments that we carried out by training a LSTM-RNN with features extracted by the VGG19 base network; as a matter of fact, we obtained poor performance, largely below those yielded by using the network operating at the frame level (for the sake of conciseness we do not report detailed information on the outcomes of this experiment).

As an alternative solution to exploit temporal dimension, we decide to consider a simpler, yet effective, strategy based on the aggregation of the decisions of single frame classifiers operating over a sliding window. In this regard, we consider two aggregation strategies, namely:

- Majority vote (MV): each frame is classified individually, then the most represented class in the window is the one assigned to the frame.
- Weighted sum (WS): the classification outputs of each class across all the frames in the window are added together, then the class with the highest score is chosen.

**Fig. 6:**
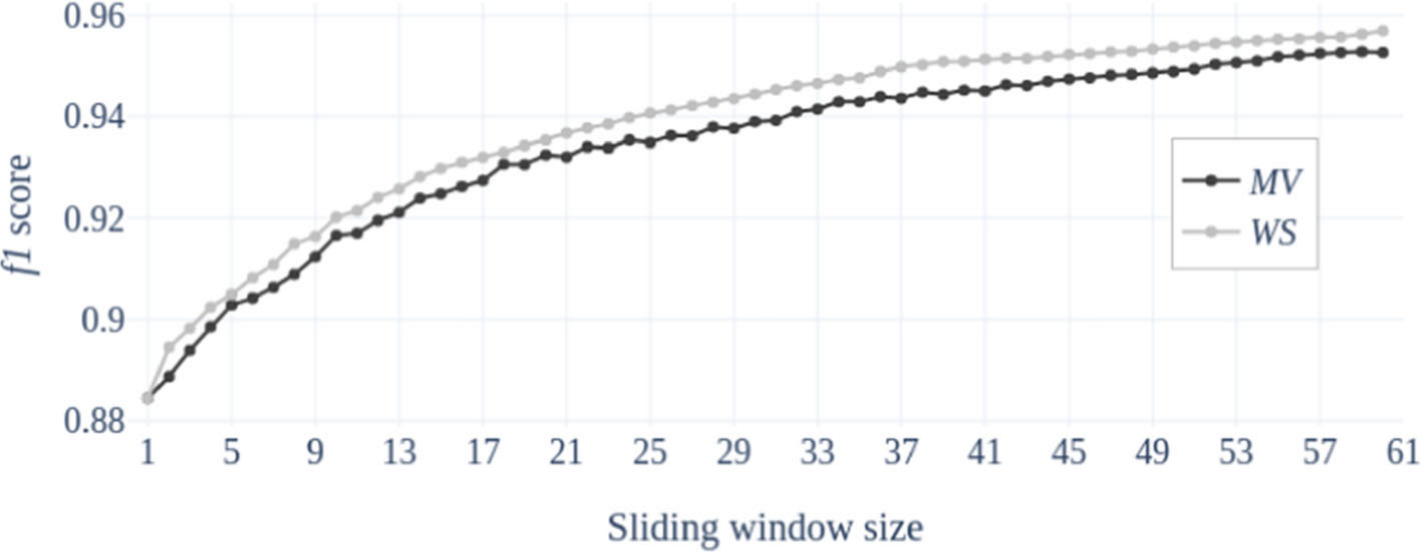
Performance assessed on the test set and reported in terms of $f_1$ score of the top performing classifier (VGG19, initialized on Imagenet, trained using mean square error loss function and Adam optimizer) when a sliding window of 15 frames (on the dataset considered in this paper 15 frames correspond to a time interval of 1 s)

The advantage of the second approach is that it does not require an additional training phase to the base single-frame classifier and does not introduce further computational burden. It only requires to set the size of the sliding window. Thus, to study the impact of this parameter on the performance, in Fig. 6 we find the curves of the performance, over the test set expressed in terms of the $f_1$ index, as a function of the sliding window size. In particular, there are included all the values from 1 to 60, where the performance with window size equal to 1 refers to the single frame classifier. The plots in Fig. 6 are all referred to the network configuration that achieves the highest performance ($f_1 = 0.884$) on the test set and highlighted in bold in Table 3.

It is immediately evident the beneficial impact of taking the decision on a frame by exploiting also the classification outputs over the preceding frames included in a sliding window. We note that the larger is the sliding window, the higher is the overall performance, which passes from $f_1 = 0.884$ with window size equal to one to $f_1 = 0.953$ adopting the MV strategy or $f_1 = 0.957$ with WS strategy, when the window size reaches the value of 60 frames. It is important to note that, since the dataset adopted for the experimental validation contains video captured at 15 fps, a window size of 60 frames corresponds to a time interval of 4 seconds that is roughly the duration of some gestures of the washing procedure (i.e., **W** and **S**). For this reason, the analysis of Figure 6 is stopped at the value of 60 frames.

Table 4, shows the confusion matrix calculated over the test set of the best performing deep neural network, according to the previous subsection. The results in the table allow us to deepen the analysis on the performance of the approach with regard to each single gesture class. We notice that for four gestures, namely **W**, **N**, **F** and **FA**, the class accuracy is above $95\%$, then there are three other gestures, **SN**, **S** and **P**, with performance around $90\%$, while only $77.4\%$ of the frames belonging to the **SH** gesture class is correctly recognized. The low performance on this class is mainly due to the erroneous attribution of $5.4\%$ and $8.8\%$ of samples of this class to the **SN** and **BH**. This confusion can be motivated by the presence of a sponge in the former or of the back of the hands in the latter as in the frames of the **SH** gesture class that the classifier is not able to discriminate correctly when taking the decision on a single frame. Similar considerations can be made on other classification errors, such as the $8.2\%$ **S** samples wrongly attributed to the **W** class.

To evaluate how the exploitation of the temporal information contributes to the mitigation of the aforementioned problems, we refer to the example cases of a sliding window with sizes 15, 30 and 60 that corresponds to a time interval of 1, 2 and 4 seconds, respectively. In particular, in Tables 5, 6, 7 and in Tables 8,  9, 10 we report the confusion matrices over the test set when using the **MV** and the **WS** aggregation rules, respectively, for the three values of the window size. As it could be expected from the results in Fig. 6, the larger is the window size the higher is the recognition rate on each single gesture class; furthermore, in all cases, the **WS** aggregation rule assures a slightly better performance compared to the majority voting. Interestingly, when using **WS** with a window of 4 seconds, we achieve almost $90\%$ accuracy on the most challenging class, that is **SH**.

**Table 4:** Confusion matrix on the test set of the VGG19 network with weights initialized on ImageNet, and trained using the mean square error loss function and the Adam optimizer

	**W**	**N**	**SN**	**SH**	**S**	**P**	**IF**	**BH**	**F**	**FA**
**W**	96.1	0.3	0.0	0.2	2.0	0.0	0.0	0.0	0.0	1.3
**N**	0.1	95.5	2.6	0.5	0.0	0.3	0.0	0.8	0.1	0.0
**SN**	0.4	0.5	88.3	6.7	0.0	2.1	0.1	0.8	1.1	0.0
**SH**	1.0	0.9	5.4	77.4	0.2	2.8	0.6	8.8	2.0	1.0
**S**	8.2	0.0	0.0	0.4	88.2	0.0	0.0	0.0	0.0	3.2
**P**	0.1	0.1	0.1	0.3	0.0	90.9	2.4	4.0	0.1	1.9
**IF**	0.7	0.6	0.0	0.6	0.0	9.6	83.3	4.3	0.8	0.0
**BH**	0.5	0.3	0.5	4.8	0.9	1.9	0.6	86.6	1.8	2.0
**F**	0.0	0.0	0.0	0.9	0.1	1.1	1.8	0.3	95.8	0.0
**FA**	1.1	0.4	0.1	0.0	0.4	0.7	0.0	2.0	0.0	95.4

Results are referred to the single frame classifier, i.e., sliding windows size equal to 1

**Table 5:** Confusion matrix on the test set of the VGG19 network with weights initialized on ImageNet, and trained using the mean square error loss function and the Adam optimizer

	**W**	**N**	**SN**	**SH**	**S**	**P**	**IF**	**BH**	**F**	**FA**
**W**	98,8	0,0	0,0	0,0	1,2	0,0	0,0	0,0	0,0	0,0
**N**	0,1	96,4	2,7	0,3	0,0	0,0	0,0	0,4	0,0	0,0
**SN**	0,0	0,2	94,4	3,5	0,0	1,2	0,0	0,0	0,7	0,0
**SH**	1,4	0,3	4,4	84,8	0,0	1,6	0,2	6,2	0,9	0,3
**S**	9,5	0,0	0,0	0,7	89,6	0,0	0,0	0,0	0,0	0,2
**P**	0,0	0,0	0,2	0,3	0,0	96,7	0,4	1,6	0,0	0,8
**IF**	0,0	0,1	0,0	0,1	0,0	7,5	88,1	3,4	0,8	0,0
**BH**	0,4	0,0	0,4	3,9	0,6	1,6	0,1	90,2	2,1	0,7
**F**	0,0	0,0	0,0	0,4	0,0	1,5	1,5	0,0	96,6	0,0
**FA**	0,3	0,1	0,0	0,0	0,0	0,8	0,0	1,1	0,0	97,7

Results are obtained setting the size of the sliding window equal to 15 and using the majority voting (MV) aggregation rule

**Table 6:** Confusion matrix on the test set of the VGG19 network with weights initialized on ImageNet, and trained using the mean square error loss function and the Adam optimizer

	**W**	**N**	**SN**	**SH**	**S**	**P**	**IF**	**BH**	**F**	**FA**
**W**	100.0	0.0	0.0	0.0	0.0	0.0	0.0	0.0	0.0	0.0
**N**	0.1	97.3	2.5	0.0	0.0	0.0	0.0	0.2	0.0	0.0
**SN**	0.0	0.2	96.5	2.3	0.0	0.8	0.0	0.0	0.2	0.0
**SH**	1.7	0.0	4.0	87.1	0.0	1.3	0.0	5.5	0.3	0.0
**S**	8.2	0.0	0.0	0.7	90.9	0.0	0.0	0.0	0.0	0.2
**P**	0.0	0.0	0.2	0.3	0.0	96.9	0.4	2.1	0.0	0.1
**IF**	0.0	0.0	0.0	0.1	0.0	5.4	92.4	2.1	0.0	0.0
**BH**	0.0	0.0	0.1	3.5	0.4	1.8	0.0	91.2	2.2	0.8
**F**	0.0	0.0	0.0	0.4	0.0	1.6	1.9	0.0	96.1	0.0
**FA**	0.0	0.0	0.0	0.0	0.0	0.8	0.0	1.0	0.0	98.3

Results are obtained setting the size of the sliding window equal to 30 and using the majority voting (MV) aggregation rule

**Table 7:** Confusion matrix on the test set of the VGG19 network with weights initialized on ImageNet, and trained using the mean square error loss function and the Adam optimizer

	**W**	**N**	**SN**	**SH**	**S**	**P**	**IF**	**BH**	**F**	**FA**
**W**	100.0	0.0	0.0	0.0	0.0	0.0	0.0	0.0	0.0	0.0
**N**	0.1	98.0	1.9	0.0	0.0	0.0	0.0	0.0	0.0	0.0
**SN**	0.0	0.2	98.2	1.2	0.0	0.4	0.0	0.0	0.0	0.0
**SH**	1.8	0.0	2.9	89.1	0.0	1.5	0.0	4.8	0.0	0.0
**S**	8.2	0.0	0.0	0.7	90.9	0.0	0.0	0.0	0.0	0.2
**P**	0.0	0.0	0.2	0.3	0.0	98.3	0.4	0.8	0.0	0.0
**IF**	0.0	0.0	0.0	0.1	0.0	3.6	95.5	0.8	0.0	0.0
**BH**	0.0	0.0	0.1	2.6	0.0	2.4	0.0	93.0	2.0	0.0
**F**	0.0	0.0	0.0	0.4	0.0	1.6	1.2	0.0	96.8	0.0
**FA**	0.0	0.0	0.0	0.0	0.0	0.8	0.0	1.0	0.0	98.3

Results are obtained setting the size of the sliding window equal to 60 and using the majority voting (MV) aggregation rule

**Table 8:** Confusion matrix on the test set of the VGG19 network with weights initialized on ImageNet, and trained using the mean square error loss function and the Adam optimizer

	**W**	**N**	**SN**	**SH**	**S**	**P**	**IF**	**BH**	**F**	**FA**
**W**	98,7	0,0	0,0	0,0	1,3	0,0	0,0	0,0	0,0	0,0
**N**	0,0	96,7	2,6	0,3	0,0	0,0	0,0	0,4	0,0	0,0
**SN**	0,0	0,2	94,5	3,6	0,0	1,1	0,0	0,1	0,6	0,0
**SH**	1,4	0,2	3,8	85,5	0,0	1,7	0,0	6,2	1,0	0,2
**S**	9,1	0,0	0,0	0,0	90,2	0,0	0,0	0,0	0,0	0,7
**P**	0,0	0,0	0,2	0,1	0,0	96,8	0,4	1,8	0,0	0,6
**IF**	0,0	0,1	0,0	0,1	0,0	6,2	90,0	2,9	0,8	0,0
**BH**	0,3	0,0	0,4	3,9	0,7	1,1	0,0	90,7	2,3	0,6
**F**	0,0	0,0	0,0	0,4	0,0	1,3	1,4	0,0	96,9	0,0
**FA**	0,0	0,0	0,0	0,0	0,0	0,7	0,0	1,0	0,0	98,3

Results are obtained setting the size of the sliding window equal to 15 and using the weighted sum (WS) aggregation rule

**Table 9:** Confusion matrix on the test set of the VGG19 network with weights initialized on ImageNet, and trained using the mean square error loss function and the Adam optimizer

	**W**	**N**	**SN**	**SH**	**S**	**P**	**IF**	**BH**	**F**	**FA**
**W**	100.0	0.0	0.0	0.0	0.0	0.0	0.0	0.0	0.0	0.0
**N**	0.0	97.3	2.4	0.0	0.0	0.0	0.0	0.3	0.0	0.0
**SN**	0.0	0.2	96.8	1.9	0.0	0.9	0.0	0.0	0.2	0.0
**SH**	1.5	0.0	3.3	88.0	0.0	1.6	0.0	5.2	0.4	0.0
**S**	7.0	0.0	0.0	0.0	92.3	0.0	0.0	0.0	0.0	0.7
**P**	0.0	0.0	0.2	0.1	0.0	96.7	0.4	2.5	0.0	0.0
**IF**	0.0	0.0	0.0	0.1	0.0	3.9	94.4	1.5	0.0	0.0
**BH**	0.0	0.0	0.0	3.6	0.6	1.1	0.0	91.7	2.5	0.7
**F**	0.0	0.0	0.0	0.4	0.0	1.4	1.6	0.0	96.6	0.0
**FA**	0.0	0.0	0.0	0.0	0.0	0.7	0.0	0.7	0.0	98.6

Results are obtained setting the size of the sliding window equal to 30 and using the weighted sum (WS) aggregation rule

**Table 10:** Confusion matrix on the test set of the VGG19 network with weights initialized on ImageNet, and trained using the mean square error loss function and the Adam optimizer

	**W**	**N**	**SN**	**SH**	**S**	**P**	**IF**	**BH**	**F**	**FA**
**W**	100.0	0.0	0.0	0.0	0.0	0.0	0.0	0.0	0.0	0.0
**N**	0.0	98.2	1.8	0.0	0.0	0.0	0.0	0.0	0.0	0.0
**SN**	0.0	0.2	98.9	0.5	0.0	0.5	0.0	0.0	0.0	0.0
**SH**	1.3	0.0	2.5	89.9	0.0	1.6	0.0	4.8	0.0	0.0
**S**	7.0	0.0	0.0	0.0	92.3	0.0	0.0	0.0	0.0	0.7
**P**	0.0	0.0	0.2	0.1	0.0	98.2	0.4	1.1	0.0	0.0
**IF**	0.0	0.0	0.0	0.1	0.0	3.2	96.3	0.5	0.0	0.0
**BH**	0.0	0.0	0.0	2.6	0.0	1.4	0.0	93.2	2.7	0.7
**F**	0.0	0.0	0.0	0.4	0.0	1.4	1.1	0.0	97.1	0.0
**FA**	0.0	0.0	0.0	0.0	0.0	0.7	0.0	0.7	0.0	98.6

Results are obtained setting the size of the sliding window equal to 60 and using the weighted sum (WS) aggregation rule

## Conclusions

In this paper, we presented an embedded system for evaluating, in real-time at what extent the medical staff is compliant with the surgical hadwashing procedure as defined by the WHO. The system exploits a deep convolutional neural network to analyze the frames captured by a depth camera. In the design of the system, we considered five well-established deep neural networks architecture (MobileNet, NASNet, ResNet50, VGG19, Xception), with four optimizers (Adam, Adadelta, SGD, RMSprop), two loss functions (mean square error and the categorical cross entropy) and two approaches for weight initialization (random and from the network trained in a different domain). We also verified that the aggregation of the decisions on a sliding window allowed us to significantly improve performance with respect to the decision taken on single frames. To this aim, in the tests we have explored both majority voting and weighted sum decision aggregation rules.

The experimental analysis was conducted using the dataset collected with the support of the medical staff of the Department of Medicine, Surgery and Dentistry - “Schola Medica Salernitana” of the University of Salerno, Italy. It included 74 video sequences, each referring to the execution of a complete hand washing procedure, and overall it comprised more than 131, 000 frames; the videos were captured at constant frame rate of 15 frames per seconds, corresponding to more than 2 hours of video footage. To the best of our knowledge, this is the first dataset in the literature dedicated to this highly specific problem in the area of gesture recognition. The dataset is made publicly available for scientific purposes upon request.

At the end of the experimental analysis, the best performing configuration was based on VGG19, initialized on ImageNet, trained using the mean square error loss function *via* the Adam optimizer; the proposed method achieved valuable performance in classifying the gestures among the 10 different classes defined by the WHO procedure, with an $F_1$ score of 0.957 by aggregating the classification outputs on window of 4 frames using a weighted sum.

We started to work of the system several months before the the outbreak of the COVID-19 pandemic. It is a preliminary system, thus presenting several limitations. Due to the relevance of the hand hygiene, we plan to extend the dataset for obtaining a more extensive and significant performance assessment of the proposed approach; this will also allow us to explore the adoption of more sophisticated network architecture specifically suited for operating on video sequences, such as the RNNs. Furthermore, we will also consider the possibility of extending the proposed method to recognize the handwashing gestures using alcohol-based solutions that can be adopted for paramedical staff, patients and visiting persons, but it may be also adopted in all the industrial sectors where careful hands hygiene is mandatory, such as like food preparation, conservation industry, restaurants.

## Footnotes

1 the dataset is available at this link https://mivia.unisa.it/datasets/
